# Paeonol eradicates biofilm in porcine-source Escherichia coli by targeting the quorum sensing system

**DOI:** 10.1186/s12917-025-05095-y

**Published:** 2025-11-04

**Authors:** Hongzao Yang, Yuan Liang, Zhuo Yang, Lin Liu, Lei Ran, Jingjing Liu, Chengjun Ma, Wei Wei, Suhui Zhang, Maixun Zhu, Hongwei Chen

**Affiliations:** 1 College of Veterinary Medicine, Southwest University, Chongqing, 402460 China; 2 Chongqing Academy of Animal Sciences, Chongqing, 402460 China; 3 Chi Institute of Traditional Chinese Veterinary Medicine, Southwest University, Chongqing, 402460 China; 4 Dazhou Key Laboratory of Animal Infectious Disease Prevention and Control, Dazhou, 636250 China

**Keywords:** Paeonol, *Escherichia coli*, Biofilm, Eradication, Quorum sensing

## Abstract

**Background:**

Natural active compounds hold significant potential to overcome biofilm-mediated resistance, offering a promising therapeutic strategy for combating bacterial biofilms resistance. The present study was designed to investigate the efficacy of paeonol in eradicating biofilms formed by porcine-derived *Escherichia coli* (strain *Ec*032), and to elucidate the underlying mechanisms of paeonol eradicating *Ec*032 biofilm.

**Results:**

The results indicated that treatment with paeonol at a concentration of 2,048 µg/mL for 3 h significantly reduced the number of viable bacteria in the mature biofilms of *Ec*032, resulting the highest biofilms eradication rate. RT-qPCR analysis suggested that paeonol might attenuate biofilms maturation by modulating the expression of quorum sensing (QS)-related and flagellum assembly-related genes. The Data Independent Acquisition (DIA) proteomic further revealed that paeonol treatment markedly inhibited flagellar motility and reduced the extracellular polysaccharide (EPS) content, leading to structural loosening of structure of the mature biofilms. Additionally, paeonol acted as a QS inhibitor (QSI), suppressing violacein in *Chromobacterium violaceum* 026 (*CV*026). Molecular docking analysis indicated that the outer membrane proteins regulator (OmpR) serve as a potential key target of paeonol.

**Conclusions:**

The research demonstrated that paeonol functions as an effective QSI, reducing biofilm biomass through downregulation of key QS and EPS matrix-associated genes and proteins, leading to effective eradication *Ec*032 biofilms. These findings provided a scientific foundation for the development of paeonol as a novel biofilm-disrupting agent and offer valuable insights for the treatment of *E.coli* biofilm-associated infections (BAI).

## Background

According to China surveillance data, the top two clinical isolated strains nationwide are *Escherichia coli* (*E.coli*) and *Klebsiella pneumonia* [1]. *E.coli* is widely present in the natural environment and a major pathogen in animal farming, posing serious risks to both livestock production and public health [2]. The increasing antibiotic resistance of *E.coli* has led to poor treatment outcomes, largely due to its ability to form biofilms. Biofilms greatly enhance bacterial resistance by employing Quorum Sensing (QS) to coordinate behavior and producing an Extracellular Polysaccharide (EPS) that provides structural protection [3]. These mechanisms render antibiotics ineffective against embedded bacteria. There is thus a critical need to develop new anti-biofilm strategies that target QS and EPS disruption to eliminate mature biofilms.

Natural active compounds offer distinct advantages in the management of infectious diseases, including holistic therapeutic effects, low risk of developing drug resistance, low cost, and acting on multiple target sites [4]. In the current “post-antibiotic era”, the application of edible and medicinal natural bioactive natural compounds—particularly in feed conversion—has become a key research focus, holding irreplaceable potential for combating Biofilm-Associated Infections (BAI) is irreplaceable. Paeonol, a major active constituent derived from moutan cortex, possesses broad-spectrum anti-inflammatory, analgesic, and antibacterial activities [5]. It has been shown to significant inhibit the growth of *Klebsiella pneumoniae* and *Enterobacter cloacae* biofilms, and also exhibits strong biofilms-eradicating capability [6]. These attributes underscore the promising potential of paeonol as a novel anti-biofilm agent, indicating considerable value for clinical applications in BAI treatment.

Nevertheless, preliminary findings by the research team suggest that paeonol exhibits notable efficacy in eradicating biofilms formed by clinical isolates porcine-derived *E.coli*, though its underlying mechanism remains unclear. This study aims to integrate phenotypic assessments of paeonol’s eradicated *E.coli* (strain *Ec*032) biofilms with Data Independent Acquisition (DIA) proteomics analysis to investigate alterations in protein expression. This objective is to preliminarily larify the mode of paeonol action, thereby providing a theoretical basis for its development as a novel biofilm-removing agent, and offering new strategies for the clinical treatment of *E.coli* BAI.

## Results

### Effects of paeonol concentrations on *Ec032*-biofilms biomass and viability

**Fig. 1:**
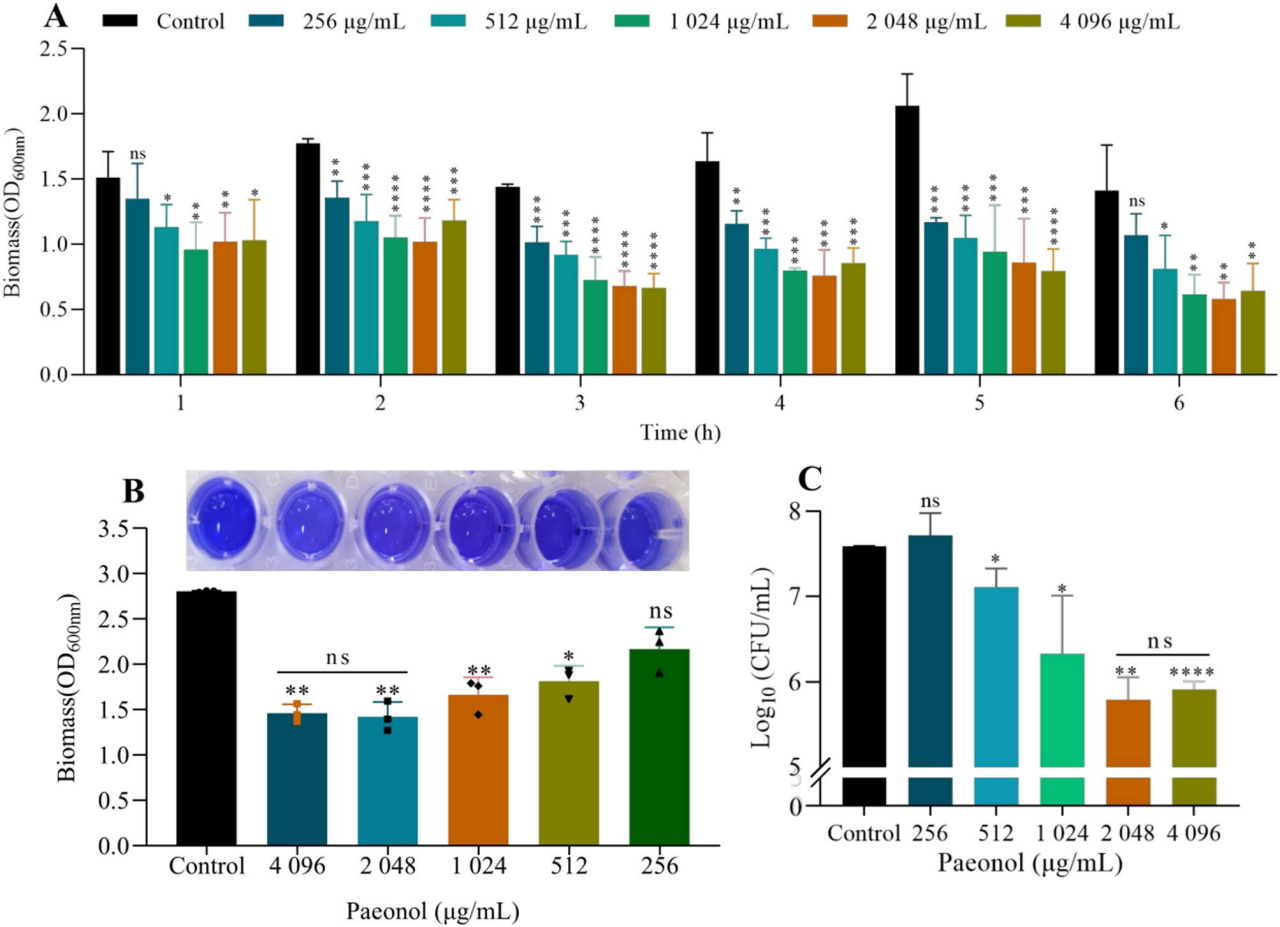
Effect of paeonol on *Ec032 * biofilm. (**A**) The biomass of *Ec*032 under the intervention of paeoniflorin at different time points was indirectly represented by measuring the OD value after crystal violet staining. (**B**) The ability of paeonol to eradicate biofilms exhibits a dose-dependent effect. (**C**) Viable bacteria of biofilm by colony counting

The MIC of paeonol against *Ec*032 was determined to be > 2048 µg/mL. Notably, even at concentrations exceeding this value, no significant antimicrobial or bactericidal effects were observed. To identify the optimal intervention conditions for paeonol against mature *Ec*032 biofilms, treatments were applied at a fixed concentration across varying time periods. The results revealed that at 2,048 µg/mL, paeonol significantly reduced *Ec*032 biofilms biomass compared to the control group, with reductions 32.66% (*P* < 0.01), 42.51% (*P* < 0.0001), 52.90% (*P* < 0.0001), 53.71% (*P* < 0.001), 58.31% (*P* < 0.001), and 56.98% (*P* < 0.01) after 1–6 h of treatment, respectively. Significantly, the biofilms clearance effect after 3 h of treatment was significantly greater than that for 2 h (Fig. 1A). Therefore, establishing 3 h as the optimal treatment duration. Although higher paeonol concentrations enhanced biofilm eradication, no statistically significant difference was observed between 4,096 µg/mL and 2,048 µg/mL (Fig. 1B). Overall, paeonol exhibited biofilm eradication in a clear dose-dependence manner within the effective concentration range.

Furthermore, viable bacterial counts within the biofilms following paeonol treatment were quantified using the plate pour method. The results show that compared to the control group, paeonol at concentrations of 4,096 µg/mL, 2,048 µg/mL, 1,024 µg/mL, and 512 µg/mL significantly reduced the number of viable bacterial in the biofilms, with reductions of 1.34, 1.45, 1.08, and 0.52 Log_10_ CFU/mL, respectively. No statistical significant difference in bacterial efficacy was observed between the 4,096 µg/mL and 2,048 µg/mL (Fig. 1C). These findings indicate that paeonol was effective against bacteria embedded in mature biofilms, with 2,048 µg/mL representing the concentration beyond which no additional significant biofilm-eradicating effect is achieved.

### Paeonol eradicated *E.coli* biofilms

**Fig. 2:**
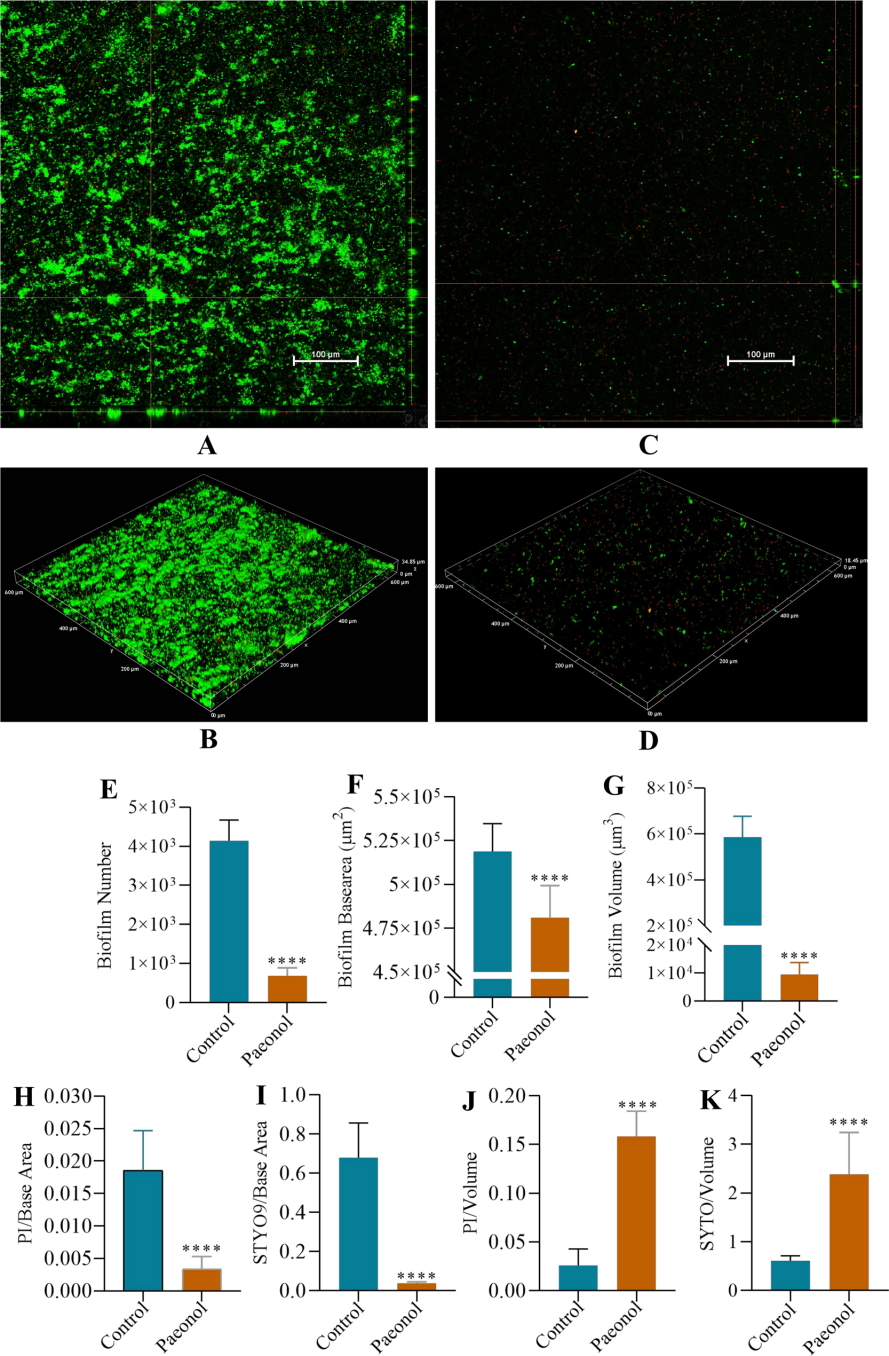
CLSM analysis of paeonol treated *Ec032* mature biofilms. (**A**) and (**B**) The 3D and orthogonal views biofilm representation in the objective of 20X by the control group. (**C**) and (**D**) The 3D and orthogonal views biofilm representation in the objective of 20X by the paeonol group. (**E**) Biofilm number. (**F**) Biofilm basearea. (**G**) Biofilm volume. (**H**) Fluorescence intensity of biofilm dead bacteria per unit area. (**I**) Fluorescence intensity of biofilm live bacteria per unit area. (**J**) Fluorescence intensity of biofilm dead bacteria per unit volume. (**K**) Fluorescence intensity of biofilm dead bacteria per unit area. Among them, the bioflm number, volume, area and fuorescence intensity were analyzed by BioflmQ software, respectively

To further validate the eradication biofilms efficacy of paeonol, CLSM was employed to visualize the architecture of preformed *Ec*032 bioflms following treatment. Biofilms were dual-stained with SYTO 9 (viable cells) and propidium iodide (PI) (dead cells) to assess cell viability and structural integrity. Representative orthogonal views of Z-stacks and 3D images (Fig. 2A-D) showed the fluorescence intensity was significantly reduced in paeonol treated bioflms compared to untreated controls. BiofilmQ software indicated significant decreases in biofilm biomass (83.56%), Biofilm basearea (7.29%) and Biofilm volume (98.01%) of *Ec*032 bioflms (Fig. 2E-G). Furthermore, the fluorescence intensity per unit base area for both PI and SYTO 9 was significantly reduced in the paeonol group, with decreases of 82.35% and 93.54%, respectively (Fig. 2H, I). In contrast, the fluorescence intensity per unit volume showed a pronounced increase: PI and SYTO 9 intensities rose by 89.90% and 71.91%, respectively (Fig. 2J, K), suggesting enhanced signal density within the remaining biofilms compartments. Collectively, these results demonstrate that paeonol treatment substantially disrupts biofilms architecture, reduces biomass and spatial extent, and alters cellular integrity. Confirming its potent biofilm-eradicating efficacy against *Ec*032 biofilms.

### Paeonol downregulated most biofilm-related genes except mqsR

**Fig. 3:**
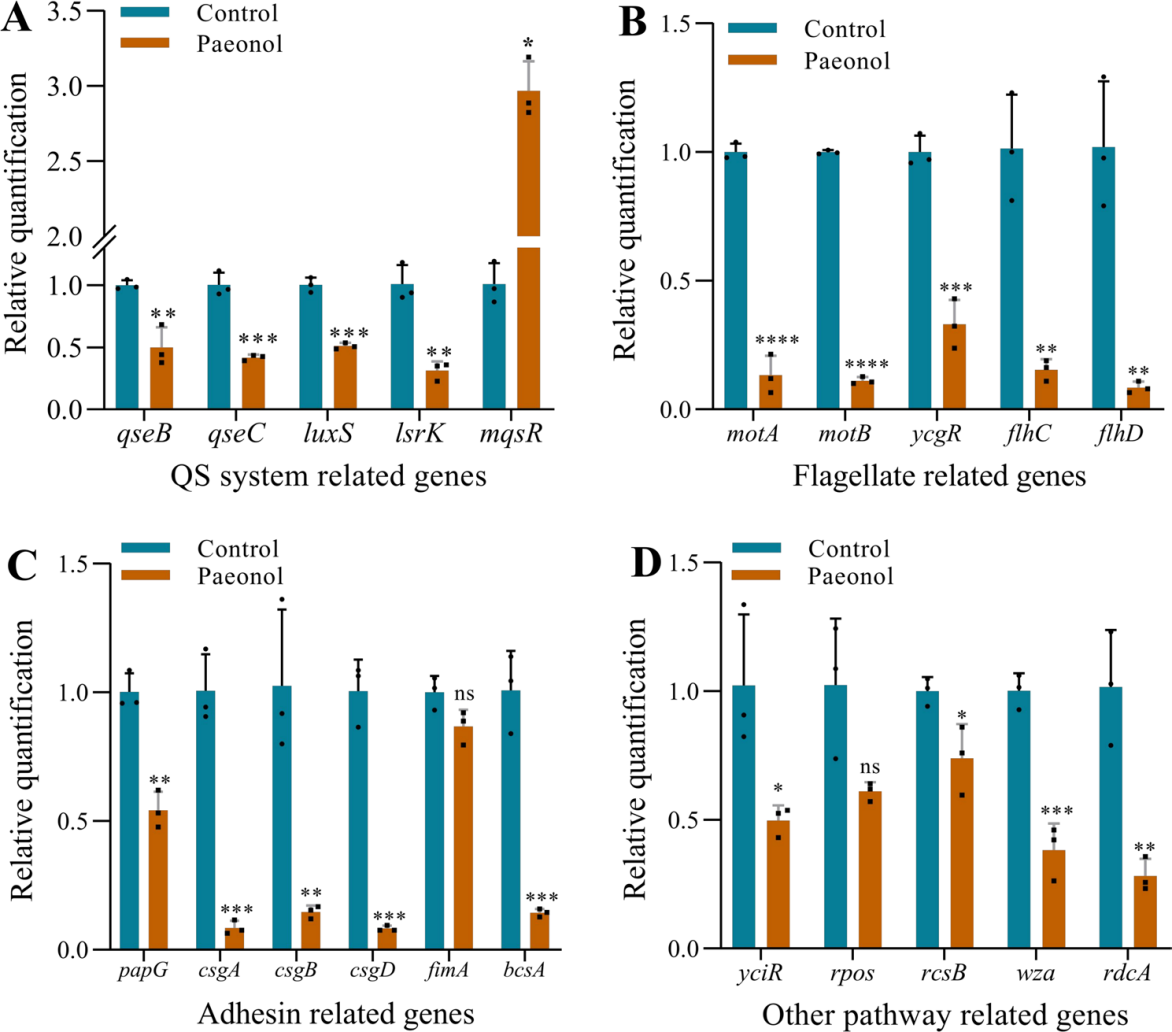
The relative transcription levels of biofilm-related genes. (**A**) QS system related genes. (**B**) Flagellate related genes. (**C**) Adhesin related genes; (**D**) Other pathway related genes

After paeonol treated, accompanied by down-regulation in the expression of QS-related genes (*luxS*, *lsrK*, *qseB*, and *qseC*), but *mqsR* was significant up-regulation (Fig. 3A). Flagellar-related genes (*flhD*, *flhC*, *motA*, *motB*, and *ycgR*) were down-regulation (Fig. 3B). Adhesion-related genes (*papG*, *csgA*, *csgB*, *csgD*, and *bcsA*) were all down-regulation, but *fimA* was not statistically significant (Fig. 3C). The transcriptional level of the EPS gene about *wza*, which was homologous to *gcfE*, was down-regulation, other related genes were also down-regulation (Fig. 3D). These findings suggest that paeonol target multiple regulatory pathways and genes involved in *E.coli* biofilms formation and maintenance, leading to the significant reduction in biofilms biomass and viability reported earlier.

### Paeonol affected the expression of biofilm related proteins by DIA analysis

**Fig. 4:**
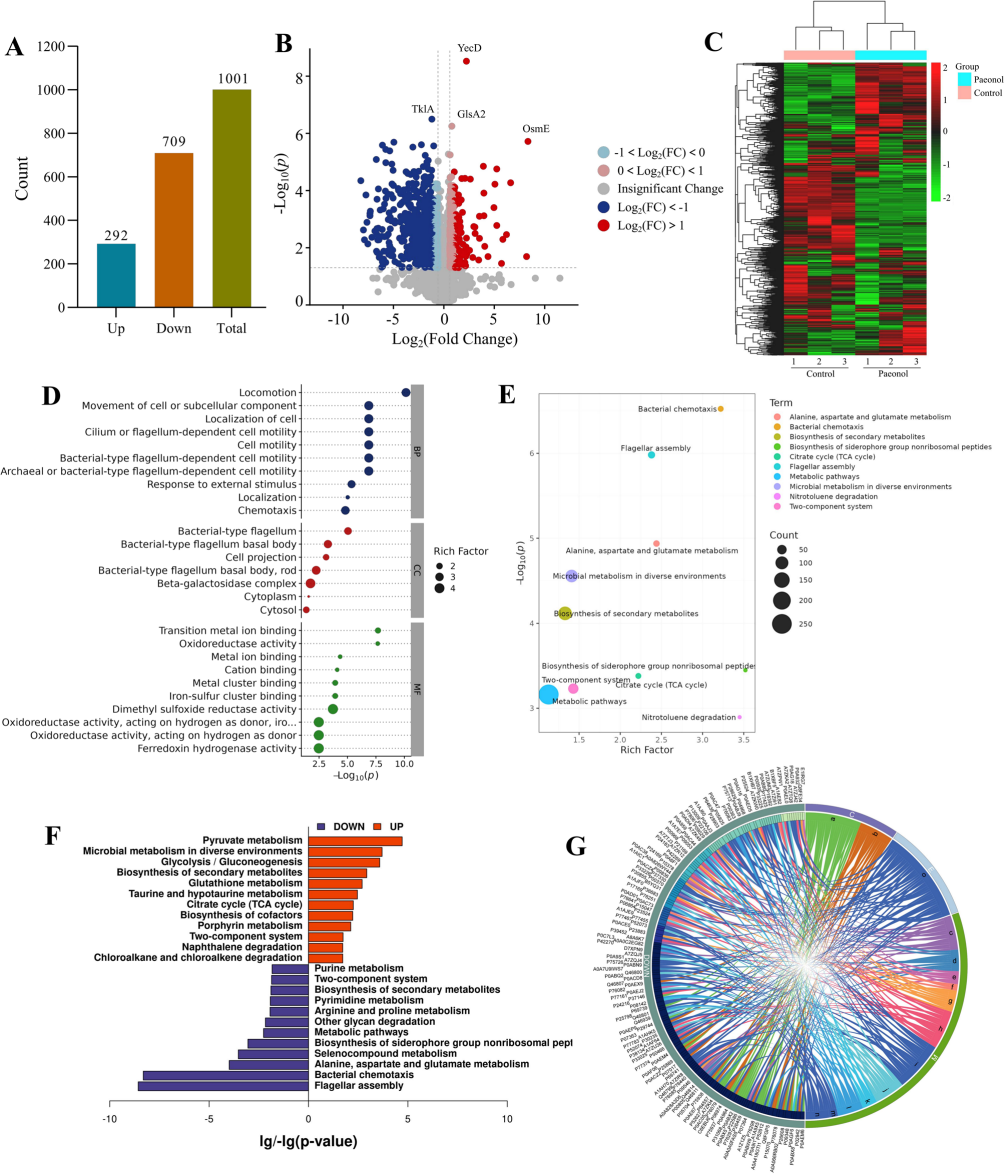
Analyzing the correlation of differential protein expression. (**A**) Number of differential proteins. (**B**) Volcano plots showed the fold change of diferential proteins in paeonol group vs. control group. The red dot is a significantly upregulated protein, the blue dots showed significantly lower expression of protein, the gray dots are non-differential proteins. (**C**) Cluster analysis of differential protein expression. (**D**) Bubble map of differential protein enrichment. (**E**) KEGG pathway enrichment of different proteins (top10). The circle size represents the quantity, and the different colors represent the individual paths. (**F**) KEGG pathway enrichment of up-regulated and down-regulated proteins. Blue columns represent down-regulated protein, and red columns represent up-regulated protein enrichment pathways. (**G**) Relationship Between Differential Proteins and Pathways: A Chord Diagram

Proteomic analysis using DIA revealed that paeonol treatment significantly altered the expression of 1001 proteins in *Ec*032 biofilms, among which 292 were up-regulated and 709 were down-regulated (Fig. 4A). Notable among the highly significant differentially expressed proteins were YecD, TklA, GlsA 2, OsmE (Fig. 4B), a clustered heatmap further illustrated the expression patterns of these proteins (Fig. 4C). To elucidate the functional implications of these changes, the GO and KEGG pathway analyses were performed. GO annotation highlighted the top ten significantly enriched terms across the three major categories: cell components (CC), molecular function (MF), and biological process (BP) (Fig. 4D). KEGG pathway enrichment analysis indicated that the top ten pathways were predominantly in nature and contained the highest number of differentially expressed proteins (Fig. 4E). Further analysis showed that down-regulated proteins were primarily enriched in flagellar assembly, whereas up-regulated proteins were linked to pyruvate metabolism. Interestingly, the two-component system (TCS) was functionally between flagellar assembly and pyruvate metabolism (Fig. 4F). A chord diagram was used to visualize the correspondence between differential proteins and relevant pathways (Fig. 4G). Biofilm-related differentially expressed proteins were systematically classified based on pathways involvement. The analysis revealed that there were mainly distributed in the QS system and bacterial motility, with the motility-related proteins, belonging to the flagellar assembly pathway (Table 1). This pathway-centric analysis offers valuable insights into the molecular mechanisms through which paeonol disrupts biofilm formation.

**Table 1:** Differentially expressed proteins associated with quorum sensing and flagellar assembly of paeonol intervention *Ec*032 bioflm

Protein	ID	Product	Log_2_ (Fold Change)	*P* < 0.05	FDR
Flagellate related the representative proteins					
FimH	P08191	Type I fibrin D-mannose-specific adhesion	−1.02649	0.034747	0.101722
FlgE	P75937	The flagellar hook protein	−2.56064	0.041467	0.114035
FlgF	P75938	The flagellar basal rod protein	−3.65048	0.014684	0.05765
FlgH	A7ZKI4	The flagellar L loop protein	−4.30144	0.000128	0.005451
FlgL	P29744	Flagellar hook-associated proteins	−2.55501	0.031210	0.094066
FlgM	P0AEM4	A negative regulator of flagellin synthesis	−3.11295	0.001333	0.014904
FliC	B7USU2	Flagellin protein	−4.15076	0.048799	0.127855
FliD	P24216	Flagellar hook-associated proteins	−2.56227	0.032770	0.097179
FliF	P25798	Flagellar M loop protein	−2.69079	0.006461	0.034971
FliH	P31068	The flagellar synthesis protein	−2.25999	0.017590	0.065617
FliI	P52612	Flagellar-specific ATP synthetase	−6.49255	0.000087	0.004638
FliM	P06974	The flagellar motor switch proteins	−5.22377	0.000139	0.005591
FliN	P15070	The flagellar motor switch proteins	−6.64995	0.003409	0.024698
YcgR	P76010	Flagellar brake protein	−3.32071	0.000310	0.007374
MotB	P0AF06	Motor protein B	−3.15661	0.005233	0.031267
CsgD	P52106	csgBAC Operon transcription regulatory protein	−2.630017	0.001212	0.014490
YdiV	P76204	Putative anti-FlhC2D4 factor	−0.699854	0.001328	0.014903
QS system related the representative proteins					
LsrA	Q8XAY7	AI-2 was input into the ATP-binding protein	−1.24599	0.017274	0.064792
LsrD	A8A068	The AI-2 input system permease protein LsrD	−1.28718	0.001764	0.017691
LsrF	A7ZLX4	3-hydroxyl-5-oxogentane phosphate-2,4-diketothioatase	−0.70379	0.008582	0.040709
LsrK	A7ZLW9	AI-2 kinase	−1.81263	0.000668	0.011202
LsrR	A8A065	The transcriptional regulator	−0.91206	0.001029	0.013768
TqsA	P0AFS5	The AI-2 transporter protein	2.5477413	0.012339	0.051382
TCS related the representative proteins					
RcsD	A0A826QV10	Phosphotransferase	0.826203	0.045135	0.120857
UvrY	P0AED5	Reaction regulator	−0.740389	0.003200	0.023728
OmpR	P0AA16	The extracellular membrane protein regulator	0.918623	0.000481	0.009347
EPS related the representative proteins					
GfcE	P0A932	A key glycosyltransferase for exopolysaccharide chain assembly.	−0.59090	0.03051	0.092794
c-di-GMP					
PdeB	P77473	Phosphodiesterase	0.611601	0.014329	0.056973
PdeL	P75800	Phosphodiesterase	−0.830624	0.002331	0.019851
Other related the representative proteins					
OmpX	P0A917	Outer membrane protein	0.926739	0.007363	0.037105
AcrD	P24177	Putative aminoglycoside efflux pump	0.595218	0.009581	0.043787
AcrE	P24180	The multidrug export protein	5.245012	0.000017	0.002923
AcrA	P0AE06	Multidrug efflux pump subunit	1.025486	0.024429	0.080365
AcrF	A0A828IPI4	Efflux pump cell membrane transporter	0.000053	6.621870	0.004262
TolA	A0A0C2K8P5	Cell envelope integrity proteins	−1.47184	0.002993	0.02285
Mcr-1	A0A0R6L508	Phosphatidylethanolamine transferase	−1.05854	0.002109	0.019068
BhsA	P0AB40	The multiple stress resistance protein	8.2029282	0.02035	0.071448
TanA	A7ZTR3	Tryptophanase	−1.560635	0.032435	0.096688
MarA	P0ACH5	Multiple antibiotic resistance proteins	5.246705	0.001668	0.017191
MdtD	A7ZNQ0	Polydrug-resistant proteins	2.96873	0.001818	0.017813
MdtQ	P33369	Multidrug-resistant outer membrane proteins	2.279707	0.016443	0.062296
MarR	P27245	Multiple antibiotic resistance proteins	3.917514	0.000014	0.002615

### Network pharmacology revealed the mechanism of paeonol eradicating biofilm

**Fig. 5:**
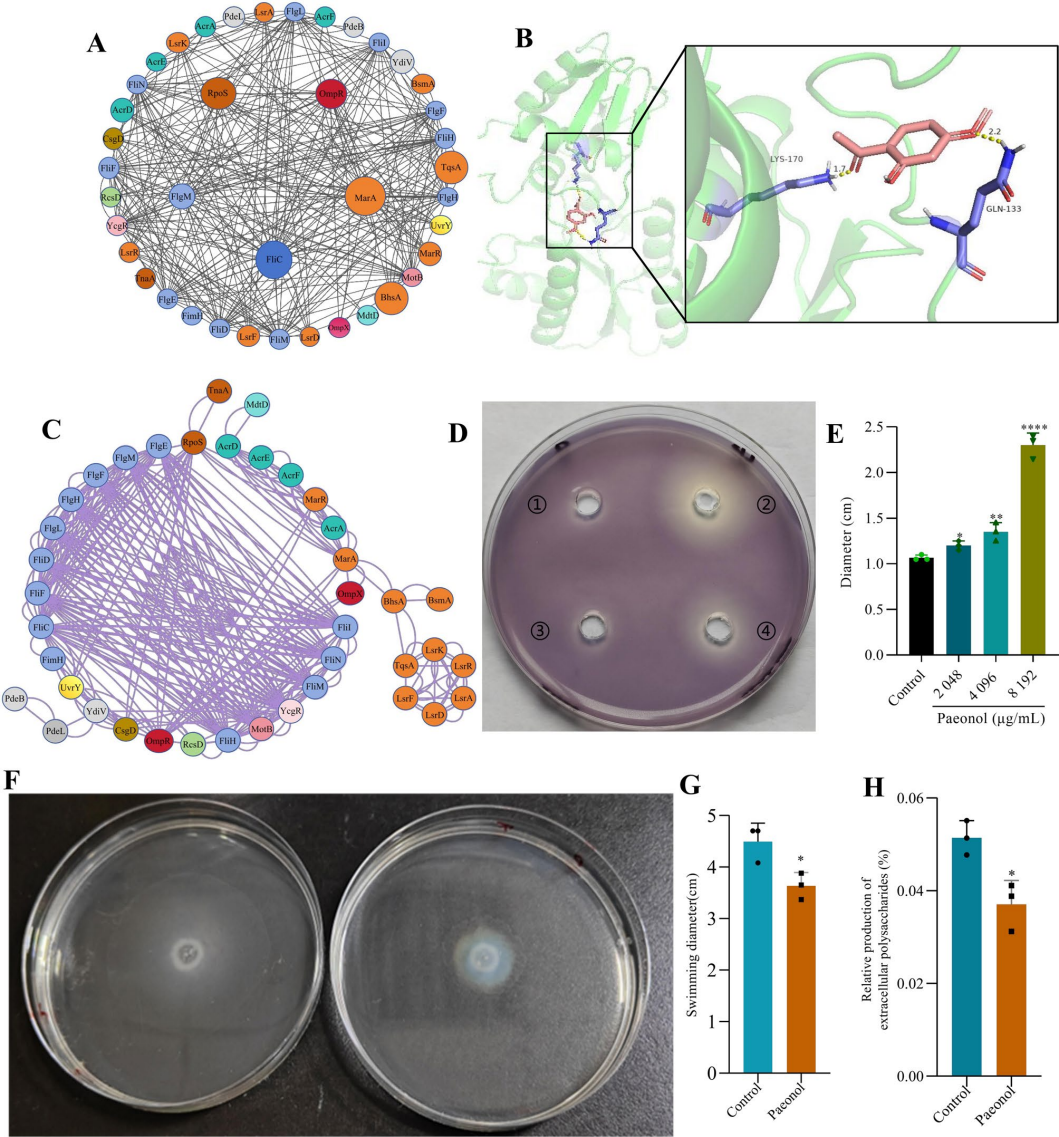
The mechanism eradicated biofilm by paeonol. (**A**) Protein-protein interaction core target screening map. (**B**) RT-qPCR validation of transcript levels for genes identified by molecular docking with paeonol. (**C**) Paeonol structural formula. (**D**) Three-dimensional interaction between paeonol and OmpR. PyMOL analyze the binding situation of receptor proteins and ligands in molecular docking, and it can mark hydrogen bonds. The yellow dotted line indicates the hydrogen bond distance or π-stacking distance, while the active ingredient is shown in red. (**E**) Protein-protein interaction network. (**F**) and (**G** The visual representation and diameter of the effect by paeonol on the *CV*026 Strain. ① represents control group. ②-④ represents paeonol concentration of 2,048 µg/mL, 4,096 µg/mL, and 8,192 µg/mL, respectively. (**H**) and (**I**) The visual representation and diameter comparison of swimming motility in biofilm-forming bacteria. (**J**) Extracellular polysaccharide content in mature biofilm

PPI analysis revealed an interaction network comprising 35 proteins in the outer circle with relatively strong connections. The inner circle consisted of core regulatory proteins, including RNA polymerase factor (RpoS, down-regulation), outer membrane protein regulator (OmpR, down-regulation), multiple antibiotic resistance protein (MarA, up-regulation), flagellar protein (FliC, down-regulation), and flagellar synthesis negative regulator (FlgM, up-regulation), all of which were further validated by RT-qPCR analysis (Fig. 5A, B). Based on these screening results and the three-dimensional structure of paeonol, molecular docking was performed, and the results were ranked in descending order according to the absolute value of binding energy (Table 2). Among all interactions, OmpR exhibited the strongest binding energy with paeonol (−5.43 kcal/mol). Therefore, the OmpR-paeonol docking complex was selected as a representative model for further analysis (Fig. 5C, D), suggesting that OmpR is a key target of paeonol. Meanwhile, 44 differentially expressed proteins were analyzed using the STRING database, The resulting network revealed close functional associations with proteins involved in bacterial flagella assembly (including FimH, FlgE, FlgF, FlgH, FlgL, FlgM, FliC, FliD, FliF, FliH, FliI, FliM, and FliN.), as well as interactions among regulatory proteins such as RpoS, OmpR, and UvrY. In addition, QS-related proteins (e.g., LsrA, LsrD, LsrF, LsrK, LsrR, and TqsA) also demonstrated significant mutual interactions (Fig. 5E). In summary, the differentially expressed proteins are primarily implicated in key biological processes including bacterial flagella assembly and QS system, and exhibit extensive functional connectivity and regulatory relationships.

**Table 2:** Binding energies of the molecular Docking by Paeonol and proteins

Proteins	Afnity (kcal/mol)
OmpR	−5.43
MarA	−4.5
FliC	−4.1
RpoS	−3.74
FlgM	−2.88

To investigate the potential QSI activity of paeonol, the bioreporter strain *CV*026 was employed monitor violacein production. Paeonol treatment resulted in a concentration-dependent increase in the inhibition zone of pigment inhibition, indicating effective suppression of violacein synthesis without inhibiting bacterial growth, thereby confirming its role as a QSI agent (Fig. 5F). Furthermore, paeonol significantly impaired bacteria flagellar motility and biofilm-forming capacity (Fig. 5G, H). Besides, the EPS content was significantly reduced by 27.9%, demonstrating that paeonol effectively decreases polysaccharide components within the EPS matrix of *Ec*032 biofilms (Fig. 5I).

## Discussion

The escalating challenge of antimicrobial resistance underscores the critical need for alternative agents against BAI. Natural product monomers, characterized by favorable biocompatibility, low-toxicity, and a reduced propensity to induce resistance, represent promising candidates for anti-biofilm strategies [6]. Among them, paeonol (C₉H₁₀O_3_) is a phenolic compound extracted from the dried root bark of the peony or the whole herb vincetoxicum pycnostelma kitag, exhibits broad pharmacological properties, including antimicrobial, anti-inflammatory, and anti-biofilm activities against pathogens such as such as *Pseudomonas aeruginosa* and *Klebsiella pneumoniae* [7–9]. In this study, crystal violet staining and CLSM demonstrated that paeonol significant eradicates *E.coli* biofilms in a dose-dependent manner. CLSM observations revealed dense and structurally intact biofilms in the control group, whereas paeonol-treated group showed markedly reduced fluorescence intensity with only residual patchy structures. Quantitative analysis using Biofilm Q software further confirmed the reduction in biofilm biomass, and the evaluation of fluorescence intensity per unit area and unit volume provided insights into the compromised viability of biofilm-embedded bacteria. These findings collectively indicate that the primary anti-biofilm mechanism of paeonol is through substantial reduction of biofilm volume and disruption of structural integrity.

Paeonol treatment significantly altered the expression of key genes involved in the bacterial QS system, including *luxS*, *lsrK*, *qseB*, and *qseC*. Specifically, *luxS*—which encodes the AI-2 synthase LuxS—and *lsrK*, responsible for AI-2 phosphorylation, were both markedly downregulated. Since AI-2 acts as a critical QS signal regulating biofilm formation and other group behaviors, these results suggest that paeonol interferes with AI-2-mediated signaling, thereby suppressing biofilm development. AI-2 is known to activate the QS regulator MqsR, which modulates biofilm architecture and influences the transcription of *qseBC* and *motAB* while repressing the major biofilm regulator *csgD* [10, 11]. Consistent with this, proteomic data revealed downregulation of QseBC, FlhDC, MotAB, and YcgR, indicating that paeonol likely restricts flagellar motility—a key factor in early biofilm formation. Intriguingly, although MqsR expression was upregulated, the transcription of its downstream targets *qseBC* and *motAB* was suppressed, suggesting that paeonol may disrupt the MqsR-mediated regulatory cascade. In contrast, the repression of *csgD* remained intact, implying that the MqsR–CsgD axis is not directly impaired. Since CsgD is critical for bacterial reattachment and biofilm stability, its downregulation may further contribute to paeonol’s anti-biofilm effects. Additionally, paeonol down regulate EPS-related protein. it downregulates bcsA, which encoding cellulose synthase—likely compromises cellulose production and curli fimbriae synthesis. The reduced transcription of waz further indicates diminished production of colanic acid, while the downregulation of *papG* implies decreased synthesis of PapG-associated adhesins and other polysaccharides such as poly-β−1,6-N-acetylglucosamine (PNAG). Collectively, these alterations lead to a structurally compromised biofilm matrix. These changes, combined with impaired flagellar motility, collectively disrupt biofilm integrity and reduce bacterial aggregation. In conclusion, paeonol exerts its anti-biofilm activity through multi-faceted mechanisms: interfering with AI-2-dependent QS signaling, disrupting MqsR-regulated pathways involved in motility and biofilm regulation, and reducing the production of key EPS components, collectively leading to the disintegration of mature biofilms.

Proteomic analysis indicates that paeonol interferes with the QS system of *E.coli*, ultimately leading to the suppression of biofilm formation. The QS-related signal transduction protein TqsA, known to modulate AI-2 transport—either promoting or inhibiting it—plays an additional role in antibiotic resistance [12]. Deletion of *tqsA* has been shown to disrupt AI-2 transport and upregulate the efflux pump genes *acrEF* [13]. Consistent with this, our results revealed upregulation of AcrE and AcrF proteins, possibly induced by TqsA activity. Notably, paeonol treatment downregulated most proteins involved in AI-2 transport and phosphorylation, in finding corroborated by RT-qPCR results. However, TqsA expression was. significantly upregulated. We hypothesize that Paeonol-induced TqsA overexpression enhances AI-2 export,, elevating extracellular AI-2 levels. Concurrently, the reduced expression of AI-2 internalization proteins LsrA and LsrD limited AI-2 uptake. Consequently, intracellular phospho-AI-2 levels became insufficient to alleviate LsrR-mediated repression of the *lsr* operon and *lsrR* itself [14]. This disruption impedes the activation of AI-2-dependent QS responses—including those necessary for biofilm formation—as the critical threshold for AI-2 uptake and downstream signaling is not attained [15]. Furthermore, using the AHL-negative biosensor strain *CV*026, which produces violacein in response to exogenous autoinducers, we confirmed that paeonol suppresses violacein synthesis, affirming its role as a QSI. This aligns with previous reports identifying paeonol as the active QSI component in moutan cortex extracts [16, 17]. Collectively, these results demonstrate that paeonol disrupts QS-mediated biofilm formation by dysregulating AI-2 transport—simultaneously promoting export and inhibiting import—thereby disturbing AI-2 sensing and downstream gene activation required for biofilm maturation.

Proteomic profiling in this study revealed a consistent downregulation of key proteins essential for flagellar assembly—including FliC, FliG, FliM, FliN, FlgE, FlgF, FlgH, FlgL, FliD, FliF, FliH, FliI, and the motility modulator YcgR. This expression profile, corroborated by phenotypic motility assays, indicates that paeonol disrupts bacterial flagellar biosynthesis and impairs swimming motility. Since functional flagella are critical for surface attachment and biofilm initiation, their compromised assembly is likely to hinder the structural integration and stability of the EPS matrix, thereby reducing biofilm development [18]. In parallel, paeonol perturbed the expression of regulatory systems involved in biofilm maturation. The BarA-UvrY TCS, which modulates fimbrial expression and bacterial adhesion, was affected through the downregulation of UvrY [19, 20]. This suggests potential impairment in type I fimbria production and initial surface attachment. Conversely, the RcsCDB phosphorelay system exhibited altered activity, RcsD was upregulated, while RcsB and RcsC remained unchanged [21]. This shift may enhance phosphosignaling through RcsCDB, ultimately influencing the expression of biofilm regulators such as csgD and flhDC, which in turn modulates curli fimbria synthesis and EPS production [22]. Additionally, the downregulation of the stationary-phase sigma factor RpoS and the biofilm master regulator CsgD further indicates that paeonol interferes with the transcriptional reprogramming required for biofilm maturation and surface reattachment. In summary, paeonol targets multiple hierarchical processes—including flagellar assembly, fimbrial production, and phosphorelay-mediated regulation—to disrupt both initial adhesion and structural maintenance of biofilms. By impairing motility, EPS composition, and regulatory circuits involving RpoS and CsgD, paeonol effectively undermines the structural integrity and developmental trajectory of *E.coli* biofilms.

Paeonol, a plant-derived phenolic compound, appears to modulate the MarRAB operon a key regulatory system involved in multidrug resistance and biofilm formation [23]. MarA, a repressor of the *marRAB* operon, is known to be bound and inactivated by certain phenolic compounds [24]. In this study, the upregulation of both MarA and MarR following paeonol treatment suggests that paeonol may act as an inducer by binding to MarR, thereby relieving its repression of the *marRAB* operon and enhancing MarA expression. As a transcriptional activator, MarA influences multiple cellular processes, including efflux pump expression and biofilm-related pathways. Elevated MarA levels are associated with reduced production of EPS components, consistent with the observed decrease in biofilm matrix integrity [25]. This aligns with previous reports demonstrating that paeonol reduces extracellular polysaccharide content in *Klebsiella pneumoniae* and downregulates GcfE-a key glycosyltransferase essential for the biosynthesis of extracellular polysaccharides in this pathogen further supporting this mechanism [6], proteomic and phenotypic analyses in the current study indicate that paeonol treatment leads to a notable reduction in colanic acid—a major EPS constituent in *E. coli*. The decline in colanic acid content likely contributes to structural loosening and reduced stability of mature biofilms, ultimately facilitating biofilm eradication. In conclusion, paeonol likely targets the MarR–MarA regulatory axis, attenuates polysaccharide synthesis (particularly colanic acid), and disrupts EPS matrix formation, collectively leading to the collapse of mature biofilm architecture.

Paeonol treatment triggered a marked upregulation of stress-responsive and multidrug efflux-related proteins in *E.coli*, including BsmA and BhsA (YcfR)—both members of the conserved DUF1471 (BhsA/McbA) protein family—as well as efflux pump components such as AcrA, MdtD, and MdtQ [26]. BsmA, encoded within the bsmABC operon, contributes to biofilm peroxide resistance and architectural integrity, while BhsA has been implicated in reducing cell aggregation and adhesion [27]. Although deletion of bsmA is associated with elevated flagellar motility and impaired microcolony formation, its overexpression under stress conditions may reflect a protective bacterial response aimed at maintaining biofilm stability [28]. Similarly, BhsA and multidrug efflux pumps such as MdtEF and AcrAB-TolC are often upregulated in response to phenolic compounds—as previously documented with gallic acid—facilitating compound extrusion and enhancing transient tolerance [29, 30]. Notably, although paeonol induced this stress and efflux reaction, our phenotypic results demonstrate that it still ultimately resulted in effective biofilm disruption and reduction, indicating that the compensatory overexpression of BsmA, BhsA, and efflux components was insufficient to confer full resistance. Thus, the observed overexpression likely represents a transient adaptive reaction rather than a complete neutralization of paeonol’s anti-biofilm effects. In conclusion, while paeonol stimulates a stress response that includes elevated expression of biofilm-associated resistance proteins and efflux systems, this response does not override its overall inhibitory impact on biofilm structural integrity and viability.

Outer membrane proteins (OMPs) are central to the virulence and pathogenesis of Gram-negative bacteria, mediating critical processes including host adhesion, invasion, and colonization [31]. In this study, paeonol was found to upregulate OmpX—a protein linked to bacterial virulence [32]. Previous reports indicate that ompX deletion attenuates flagellar gene expression (e.g. flhDC), impairs internalization into host cells, and reduces colonization capacity [33]. Interestingly, although OmpX was upregulated under paeonol treatment, flagellar motility was significantly inhibited, suggesting that paeonol may override or decouple the usual virulence regulation mediated by OmpX, ultimately contributing to reduced biofilm formation. Furthermore, the expression of the key two-component system response regulator OmpR was elevated, while its downstream biofilm master regulator CsgD and adhesion-related protein FimH were downregulated. This expression pattern implies that OmpR may be activated—possibly through phosphorylation—as part of a bacterial compensatory response to maintain partial expression of virulence factors such as curli fimbriae [34, 35]. Nevertheless, the net effect of paeonol treatment was a clear reduction in biofilm integrity and adhesion capacity. Molecular docking results confirm that OmpR serves as a direct target of paeonol, with high binding affinity and stable interaction. This binding likely disrupts OmpR’s regulatory function, leading to downstream suppression of key virulence and biofilm-related pathways, consistent with observed transcriptional downregulation of csgD, fimH, and flagellar assembly genes. These findings collectively demonstrate that paeonol acts through specific inhibition of OmpR-mediated signaling to attenuate biofilm formation and virulence in E. coli. Given the robust in vitro and in silico evidence, paeonol represents a highly promising anti-biofilm agent. Nevertheless, further in vivo validation and prospective clinical studies remain essential to fully assess its therapeutic potential.

## Conclusion

In conclusion, paeonol effectively eradicates mature biofilm of *E.coil* by inhibiting bacterial motility, disrupting EPS synthesis, and interfering with QS system. Paeonol directly targets OmpR, a key regulatory protein, leading to reduced biofilm formation and structural integrity. These findings support paeonol’s potential as a natural anti-biofilm agent, though further studies are needed to confirm its efficacy in vivo.

## Methods

### Bacterial strains, culture conditions

The *Escherichia col*i strain *Ec*032 used in this study was isolated and identified from a pig farm located in Chongqing, China. *Chromobacterium violaceum* 026 (*CV*026) was purchased from Beijing Bio-Bridge Biotechnology Co., Ltd, and was used to elucidate the quorum sensing inhibition (QSI) assays.

The *Ec*032 strain was initially resuscitated and cultured overnight at 37℃ in Luria-Bertani (LB) broth under aerobic conditions. A single colony was then selected and inoculated into 5 mL of mueller hinton (MH) broth, followed by incubation in a shaking incubator at 37 °C with agitation 220 rpm/min to ensure consistent aeration and promote aerobic growth. The bacterial suspension at the logarithmic phase was adjusted to an OD_*600*nm_ of 0.1 using fresh MH broth and subsequently diluted 100-fold to achieve a working concentration of approximately 10⁶ CFU/mL, which was confirmed by plate counting.

The frozen stock of *CV*026 was removed from storage and thawed. A volume of 100 µL was transferred into LB broth containing kanamycin (KAN) and incubated overnight in a shaking incubator at 28 °C with a speed of 220 r/min to ensure consistent aeration and promote aerobic growth. A small amount of the activated bacterial suspension was then streaked onto a KAN agar plate in three sections and incubated at 28 °C for 16–18 h in an inverted position.

### Preparation paeonol solutions and determination of Minimum Inhibitory Concentration (MIC)

Paeonol was purchased from Sichuan Hengruitongda Biotechnology Co., Ltd., with a purity of 98.00%. Paeonol was dissolved in dimethyl sulfoxide (DMSO) to prepare a stock solution at a concentration of 204.8 mg/mL. This stock solution was then diluted to concentrations of 102.4 mg/mL, 51.2 mg/mL, 25.6 mg/mL, and 12.8 mg/mL. Afterward, these solutions were further diluted 50-fold with MH broth to obtain working concentrations of 4,096 µg/mL, 2,048 µg/mL, 1,024 µg/mL, 512 µg/mL, and 256 µg/mL, respectively. The solutions were then sterilized by filtration through a 0.22 μm pore-sized membrane filter and stored for subsequent use.

The minimum inhibitory concentration (MIC) of paeonol against *Ec*032 was determined using the broth microdilution method according to CLSI guidelines (CLSI, 2023) with minor modifications [36]. Briefly, adding 40 µL of the paeonol concentration and an equal volume of working bacterial suspension into rows 2–8 of a 96-well polystyrene microtiter plate. An equal volume of the working bacterial suspension (approximately 10⁶ CFU/mL in Mueller-Hinton broth) was added to each well containing paeonol, as well as to the positive control well. The positive control consisted of MH broth with 2% DMSO and bacterial inoculum, while the negative control contained only MH broth and corresponding drug concentration without bacteria. The plate was incubated statically under aerobic conditions 37 °C for 16–18 h and observe the results.The MIC was defined as the lowest concentration of paeonol that completely inhibited visible bacterial growth.

### Biomass and bioflm bacterial assays

Mature biofilms of *Ec*032 were established according to a standardized static biofilm formation assay with slight modifications [37]. Briefly, a single colony of *Ec*032 was inoculated into MH broth and aerobically cultured at 37 °C with shaking at 220 rpm until the logarithmic growth phase was reached. The bacterial suspension was then adjusted to an optical density (*OD*_*600 nm*_) of 0.1, corresponding to approximately 10⁸ CFU/mL, using fresh MH broth. A volume of 100 µL of this suspension was aliquoted into each well of a sterile 96-well polystyrene microtiter plate. The plate was incubated statically under aerobic conditions at 37 °C for 24 h to allow mature biofilm development.

Mature *Ec*032 biofilms were treated with paeonol to evaluate its eradication efficacy. Following the formation of mature biofilms, the bacterial suspension was carefully aspirated, and the wells were gently washed three times with phosphate-bufered saline (PBS) to remove non-adherent cells. Subsequently, 100 µL of paeonol solution, prepared in MH broth (containing 2% DMSO) was added to each test well. An equal volume of MH broth containing 2% DMSO was used as the vehicle control. The plate was then incubated under static, aerobic conditions at 37 °C for 3 h to allow interaction between paeonol and the biofilm.

The biomass of mature biofilms were quantified using crystal violet staining. After paeonol treatment, the supernatant was carefully removed, and the biofilms were gently washed twice with PBS. The adherent biofilms were fixed with 100% methanol for 10 min and air-dried. Subsequently, the biofilms were stained with 0.04% crystal violet solution for 20 min, followed by rinsing with PBS to remove unbound dye. The bound crystal violet was solubilized with 33% glacial acetic acid, and the absorbance was measured at 600 nm. For quantification of viable biofilm-embedded bacteria, the biofilm in parallel wells were disrupted using 0.1% Triton X-100, serially diluted tenfold in PBS, and plated onto tryptic soy agar (TSA). The plates were incubated aerobically at 37 °C for 12 h, after which CFU were enumerated.

### Confocal Laser Scanning Microscopy (CLSM)

The architectural integrity and viability of biofilms were examined using CLSM. Mature biofilms, grown under controlled conditions, were divided into control and paeonol-treated groups. Each biofilm was washed with 0.9% NaCl and stained for 20 min at room temperature in the dark using a Filmtracer™ LIVE/DEADTM Biofilm Viability kit. After staining, the samples were gently rinsed with PBS to remove excess dye. Imaging was performed with a CLSM system equipped with a Plan-Apochromat 63×/1.40 oil objective lens. Signals were recorded using the green (SYTO 9, 488 nm) and red (PI, 561 nm) channels. Images were acquired using ZEN (black edition) software. The bioflm number, volume, area and fluorescence intensity was performed using the BiofilmQ analysis platform.

### Quantitative Real-Time PCR (qRT-PCR)

After co-incubating the constructed mature biofilms of *Ec*032 with paeonol for 3 h, the supernatant was discarded, and the mature biofilms were gently washed 2 to 3 times with PBS, total RNA was extracted from culture mature biofilms by Trizol reagents. Then the cDNA is generated using PrimeScript™ RT reagent Kit with gDNA Erase (Takara Co.,Ltd, China). To determine the mechanism by which paeonol eradicates the mature biofilms of *Ec*032, RT-qPCR technology was used to analyze the changes in the expression levels of related genes. According to the NCBI, we designed amplification primers for the qPCR of the test genes (Table 3) (Beijing Tsingke Biotech Co., Ltd.). Then each reaction was performed three times in duplicate using ABI 7500 Fast Real-Time PCR system (Applied Biosystems, USA). Each 20 µL reaction mixture contained 10 µL of 2× SYBR Green Pro Taq HS Premix, 2 µL of cDNA template, 0.4 µL of each forward and reverse primer (10 µM), and 7.2 µL of ddH_2_O. The thermal cycling protocol was carried out as follows: initial denaturation at 95 °C for 30 s; followed by 40 cycles of denaturation at 95 °C for 10 s and annealing/extension at 60 °C for 34 s. Melting curve analysis was subsequently conducted with the following steps: 95 °C for 15 s, 60 °C for 1 min, and then a gradual increase to 95 °C with continuous fluorescence measurement to verify amplicon specificity. The reaction without template and the negative reference samples were used as negative controls. Raw data is exported to GraphPad Prism for analysis. At the same time, relative mRNA levels were determined using the comparative Ct method and normalized against *gapA* mRNA, so as to calculate the relative mRNA level of by ^ΔΔ^ct method.

**Table 3:** Gene primer sequences in this study

Primer	Forward primer(5’→3’)	Forward primer(5’→3’)
*gapA*	GTTGTCGCTGAAGCAACTGG	CGATGTCCTGGCCAGCATAT
*mqsR*	AAAACTTGTCAATGCCGGGC	CCTGTAACAAGCCTGGGTCT
*rdcA*	GCGTTGTGCGTGAGACTTTT	GACCTGCTCTGCCAGAACTT
*csgD*	GATTACCCGTACCGCGACAT	GCGTAATCAGGTAGCTGGCA
*csgA*	TCTGGGCAGGTGTTGTTCCTC	CCGCCATGCTGGGTAATAT
*csgB*	AGTGCCAACGATGCCAGTAT	TCACGCGAATAGCCATTTGC
*motA*	CGCCGAAACCAGCAAATGA	TCCTCGGTTGTCGTCTGTTG
*motB*	TGACTGCGATGATGGCCTTT	CCCCTGGCTTTGGGTGTAAT
*fimA*	TTGTTCTGTCGGCTCTGTCC	ACTGGTTGCTCCTTCCTGTG
*papG*	TTCGCATCGTGAAACAGCAC	TACGTTTCGCTTCCATGGCT
*luxS*	TTGGTACGCCAGATGAGCAG	ACGTCACGTTCCAGAATGCT
*qseB*	ATTGGCGACGGCATCAAAAC	CACGCTGACCTTTTTCTCGC
*qseC*	CGTGACCCTGACTCGGAAAA	TTCGGTTTGCACTTTCAGCG
*lsrK*	CAGATTACTTTGGCTGGCGC	CGTAGGCCAGCCATATCCAG
*rcsB*	ATCAGTGCTGGTGGTTACGG	TCAGCAGGGCGATATCGTTC
*flhC*	ATGCTGCCATTCTCAACCATAT	GCTTGTGGGCACTGTTCAAG
*flhD*	TCCGCTATGTTTCGTCTCGG	ATCGTCAACGCGGGAATCTT
*yciR*	GTCATTGGGCAAAGCGTGTT	TTGCGAAACAGAAACAGCCG
*rpoS*	AGCTGAACGTTTACCTGCGA	TGTCCAGCAACGCTTTTTCG
*Wza*	GACTTTAGCGGCATGACCCT	CGTGGCATCGGACATATCCA
*ompR*	TGCCCGTGGTCGTGAATATT	GCGAAATCTGCACGTCGAT
*marA*	TGAAGGAAAGTAACGAGCCG	ATTCGCCCTGCATATTGGTC
*fliC*	GCGCGAAGTTAAACACCACG	CGGTGACTTTATCGCCATTCC
*rpoS*	CTATTTGGGAGAGATTGGCTATTCGC	CCAGACCACGATTGTTGTAACGGC
*flgM*	TCGCCTCTGAAGCCTGTA	CGTTACGAATCGCCAGTT

### Data Independent Acquisition (DIA) proteomics technology and network pharmacological analysis

Using a 6-well cell culture plate to construct a biofilm in vitro, administer paeonol for 3 h, and simultaneously set up a control group. Each experimental group collected three independent replicates of samples. Biofilms were collected, and proteins were extracted, reduced, alkylated, and digested with trypsin to generate peptides for analysis. and sent to the company for sequencing (Shanghai Baipu Biotechnology Co., Ltd). Digested peptides were separated by nano-liquid chromatography and analyzed using a timsTOF Pro 2 mass spectrometer operating in DIA mode. All mass spectrometry data were merged using the DIA-NN software to complete the database search, and protein DIA quantitative analysis of DIA mass spectrometry data. The database was used Uniprotkb-*Escherichia coli* [562]−1129302-20231007.fasta, containing 1,129,302 protein entries, downloaded in October 2023. The analysis was performed using DIA-NN 1.8.1 software. The organized genes related to the biofilm were imported into STRING (https://string-db.org/) to obtain a protein-protein interaction (PPI) network diagram. The obtained images were analyzed and integrated using Cytoscape 3.9.1 software. The plugin CentiScaPe 2.2 was used to analyze the network topology based on the Betweenness and degree values, to select key targets of paeonol acting on the biofilm.

For the molecular docking part, the open source software AutoDock1.5.7 was used for docking and data processing, and Pymol was used to process the docking result files and export the pictures. The best-matched 3 D structure was selected in the following databases based on the proteomics number. The 3D structure files of the target proteins were obtained from RCSB PDB database (https://www.rcsb.org/), Deep Mind database (https://alphafold.ebi.ac.uk/), SWISS-MODEL database (https://swissmodel.expasy.org/repository/), the 3D structure of paeonol was obtained from the Pubchem database (https://pubchem.ncbi.nlm.nih.gov/).

### Quorum Sensing Inhibitor (QSI) test

The bioreporter strain *CV*026 was cultured overnight and adjusted to an OD_*600 nm*_ of 1.2–1.3, The bacterial suspension was then mixed at a 1:10 ratio with molten LB agar containing Kanamycin (KAN) (20 µg/mL) and N-[(3 S)-Tetrahydro-2-oxo-3-furanyl]butanamide (C4-HSL) (10 µM/mL). The mixture was poured into sterile Petri dishes. After solidification, wells were created in the agar using sterile oxford cups. Then, 20 µL paeonol solution was added into each well, while an equal volume of 2% DMSO in LB broth was used as the vehicle control. Following 10 min drying period, the plates were inverted and incubated at 28 °C for 18–20 h. Violacein inhibition zones were subsequently observed and measured.

### Swimming test

Swimming motility assays were performed as previously described with minor modifications [38]. Briefly, swimming agar plates were prepared by dissolving 1.5 g tryptone, 1 g yeast extract, 1 g NaCl, and 0.45 g bacterial agar in 150 mL purified water. The medium was sterilized by autoclaving at 121 °C for 20 min, mixed thoroughly while cooling, and dispensed intosterile Petri dishes (approximately 20 mL per plate). The plates were dried in a laminar flow hood prior to use. Log-phase bacterial cultures were concentrated and 2 µL of suspension was carefully spotted onto the center of each swimming plate. After air-drying for 10–15 min, the plates were incubated at 37 °C for 6–8 h. Motility was assessed by measuring the diameter of the bacterial migration zone. Each experiment was independently repeated three times.

### Assays of Exopolysaccharides (EPS)

The EPS content of biofilms was quantified using a ruthenium red-based colorimetric assay, as previously described with slight modifications [39]. Following paeonol treatment, mature biofilms in 96-well plate were divided into the paeono-treated and the control groups. The supernatant was carefully aspirated, and the biofilms were gently washed three times with PBS to remove non-adherent cells and residual medium. Then, 110 µL of 0.01% (w/v) ruthenium red staining solution was added to each well, with blank control wells containing an equal volume of staining solution without biofilms. The plate was incubated at 37 °C for 60 min. After incubation, the unbound was carefully transferred to a new 96-well plate, and the absorbance was measured at OD_*450nm*_. The EPS content was calculated using the formula: EPS content = [OD_*450 nm*_ (blank control group) -OD_*450nm*_ (sample group)]× bacterial count.

### Statistical analyses

All experimental results were replicated three times. Data was analyzed by GraphPad Prism 8.0 software, and the statistical analysis was performed according to Student’s T-tests. Analysis of variance was used for statistical analyses. Signifcant diferences were indicated as *(*P* < 0.05), **(*P* < 0.01), ***(*P* < 0.001), ****(*P* < 0.0001), respectively. Not Statistically Significant acronym for “ns”.

## Data Availability

The original contributions presented in the study are included in the article, further inquiries can be directed to the corresponding author.
